# Human adaptation to high altitude: acclimatization and reversibility of haemodynamics

**DOI:** 10.1093/nsr/nwaf203

**Published:** 2025-05-21

**Authors:** Yifan Xu, Qiaoyi Hua, Wu Chen, Jicheng Gong, Xin Meng, Teng Wang, Yiqun Han, Yi Zhang, Xudong He, Chunxiang Ye, Pubu Dunzhu, Cencen Wu, Yuanyuan Fan, Lingyun Zu, Tong Zhu

**Affiliations:** SKL-ESPC & SEPKL-AERM, College of Environmental Sciences and Engineering, Institute of Tibetan Plateau, and Center for Environment and Health, Peking University, China; TUWAS-AEEEH, Xizang University, Lhasa, China; SKL-ESPC & SEPKL-AERM, College of Environmental Sciences and Engineering, Institute of Tibetan Plateau, and Center for Environment and Health, Peking University, China; SKL-ESPC & SEPKL-AERM, College of Environmental Sciences and Engineering, Institute of Tibetan Plateau, and Center for Environment and Health, Peking University, China; SKL-ESPC & SEPKL-AERM, College of Environmental Sciences and Engineering, Institute of Tibetan Plateau, and Center for Environment and Health, Peking University, China; TUWAS-AEEEH, Xizang University, Lhasa, China; SKL-ESPC & SEPKL-AERM, College of Environmental Sciences and Engineering, Institute of Tibetan Plateau, and Center for Environment and Health, Peking University, China; SKL-ESPC & SEPKL-AERM, College of Environmental Sciences and Engineering, Institute of Tibetan Plateau, and Center for Environment and Health, Peking University, China; Environmental Research Group, MRC Centre for Environment and Health, Imperial College London, UK; SKL-ESPC & SEPKL-AERM, College of Environmental Sciences and Engineering, Institute of Tibetan Plateau, and Center for Environment and Health, Peking University, China; Medical College of Xizang University, Lhasa, China; SKL-ESPC & SEPKL-AERM, College of Environmental Sciences and Engineering, Institute of Tibetan Plateau, and Center for Environment and Health, Peking University, China; TUWAS-AEEEH, Xizang University, Lhasa, China; Dingri County Central Hospital, Xigaze, China; Department of Cardiology and Institute of Vascular Medicine, Peking University Third Hospital; State Key Laboratory of Vascular Homeostasis and Remodeling, Peking University; NHC Key Laboratory of Cardiovascular Molecular Biology and Regulatory Peptides, Peking University; Beijing Key Laboratory of Cardiovascular Receptors Research, China; Department of Cardiology and Institute of Vascular Medicine, Peking University Third Hospital; State Key Laboratory of Vascular Homeostasis and Remodeling, Peking University; NHC Key Laboratory of Cardiovascular Molecular Biology and Regulatory Peptides, Peking University; Beijing Key Laboratory of Cardiovascular Receptors Research, China; Department of Cardiology and Institute of Vascular Medicine, Peking University Third Hospital; State Key Laboratory of Vascular Homeostasis and Remodeling, Peking University; NHC Key Laboratory of Cardiovascular Molecular Biology and Regulatory Peptides, Peking University; Beijing Key Laboratory of Cardiovascular Receptors Research, China; SKL-ESPC & SEPKL-AERM, College of Environmental Sciences and Engineering, Institute of Tibetan Plateau, and Center for Environment and Health, Peking University, China; TUWAS-AEEEH, Xizang University, Lhasa, China

The attraction of high-altitude (HA) destinations, i.e. elevation of ≥2500 m above sea level (ASL), has significantly increased due to tourism and work purposes. Over 100 million individuals worldwide travel to HA regions annually [1]. In China, ∼51 million people travelled to the Qinghai-Tibet Plateau (average elevation >4000 m ASL) in 2017 [2]. The HA environment is predominantly characterized by hypobaric hypoxia, along with low temperatures and high ultraviolet radiation [3]. Various studies have investigated acute mountain sicknesses (AMS) and its pathophysiological mechanisms [4,5]; however, it remains largely unknown whether these acute responses could attenuate and acclimatize following prolonged exposure to HA environments. Furthermore, de-acclimatization is a reversal of HA physiological adaptations at sea level (SL), posing potential health risks [6]. It is essential to understand the acclimatization to HA and recovery after descent, as well as the underlying biological mechanisms, to protect individuals travelling to HA for extended periods.

Haemodynamics play a fundamental role among physiological responses to HA, being closely related to blood flow and oxygen transport [5,7]. While several studies have investigated haemodynamic changes from SL to HA [7], they either focus solely on the acute phase of HA exposure or lack comprehensive

analysis spanning from HA exposure to re-acclimatization at SL. Furthermore, the molecular mechanisms underlying acclimatization and subsequent reversibility remain inadequately elucidated. Transcriptomic analysis, which enables unbiased RNA profiling, may help to address these limitations.

By conducting a cohort study (Fig. 1a) that integrated detailed haemodynamic measurements with cutting-edge transcriptomic analysis, we investigated haemodynamic acclimatization to HA exposure and its reversibility after descending to SL, as well as the underlying mechanisms. Details of the methods are reported in the Supplemental Materials online. The study was approved by the Institutional Review Board of the Health Science Center of Peking University (approval no. IRB00001052-19062), and written informed consent was obtained from all participants.

**Figure 1.**
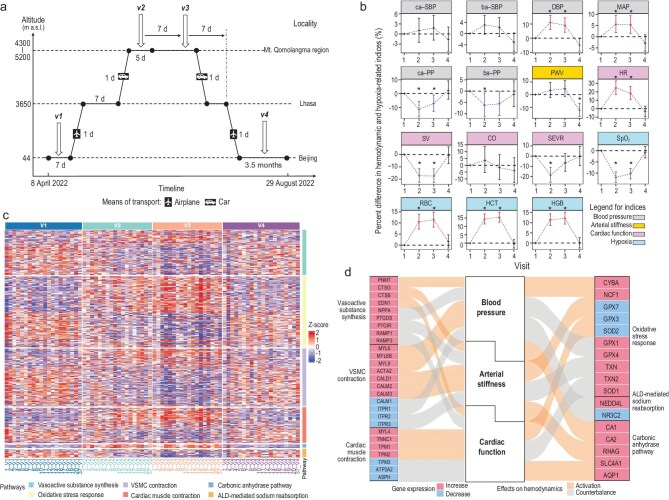
(a) Timeline of the study. Participants finished the first clinical visit (baseline, denoted as v1) at Beijing and, in the following week, travelled by air to Lhasa, where they stayed for a week. Later, they went to the Mt. Qomolangma region by car and finished the second clinical visit (denoted as v2) within 5 days upon arrival; the third clinical visit (denoted as v3) was conducted after a 7-day interval. In the following week, participants returned to Beijing and finished the last clinical visit (denoted as v4) within 3.5 months. (b) Differences in haemodynamic and hypoxia-related indices at four clinic visits. Significant positive and negative differences with a *P*-value of <0.05 are shown in red and blue, respectively. Differences with *FDR*_B-H_ < 0.05 are marked with asterisks. (c) The expression levels of genes involved in haemodynamics-regulating pathways at four clinic visits. The pathways include vasoactive substance synthesis, oxidative stress response, VSMC contraction, cardiac muscle contraction, carbonic anhydrase pathway and aldosterone-mediated sodium reabsorption. The gene-expression levels were normalized as a subject-wise Z score, i.e. each gene was individually normalized for each subject across their four clinical visits, with a mean of zero and a standard deviation of 1. (d) Adaptive responses of the haemodynamics-regulating pathways to HA exposure and their effects on haemodynamics. Genes are linked to haemodynamic modulations including blood pressure, arterial stiffness and cardiac function. Genes in red and blue were upregulated and downregulated at HA, respectively. Bands in yellow and grey indicate the activation and counterbalance effects of genes to the haemodynamic modulations, respectively. ca-SBP, central aortic systolic blood pressure; ba-SBP, brachial artery systolic blood pressure; DBP, diastolic blood pressure; MAP, mean arterial pressure; ca-PP, central aortic pulse pressure; ba-PP, brachial artery pulse pressure; PWV, pulse wave velocity; HR, heart rate; SV, stroke volume; CO, cardiac output; SEVR, subendocardial viability ratio; SpO_2_, blood oxygen saturation; RBC, red blood cell; HCT, haematocrit; HGB, haemoglobin; VSMC, vascular smooth muscle cell; ALD, aldosterone.

A total of 21 participants (average age: 32.5 years) completed 83 clinic visits (Table S1). During their stay at HA, 81% of the participants reported symptoms of AMS, 48% of the participants reported using oxygen and 14% used medication to alleviate symptoms.

Table S2 and Fig. 1b present the levels and changes in haemodynamics and hypoxia-related indices at different altitudes, respectively. During the initial transition from SL to HA (i.e. v1 to v2),

most haemodynamic indices exhibited significant changes compared with the baseline values measured at SL (v1). Diastolic blood pressure (DBP) and mean arterial pressure (MAP) significantly increased by 11.0% (95% confidence interval: 6.1% to 16.1%) and 5.6% (1.8% to 9.6%), respectively. Conversely, central aortic pulse pressure (ca-PP) and brachial artery pulse pressure (ba-PP) exhibited significant decreases (Table S3). Brachial artery systolic blood pressure (ba-SBP), central aortic systolic blood pressure (ca-SBP) and pulse wave velocity (PWV) (a measure of arterial stiffness) showed a nonsignificant upward trend. Cardiac-function indices, stroke volume (SV) and subendocardial viability ratio (SEVR) significantly decreased by 17.2% (11.5% to 22.6%) and 18.6% (10.8% to 25.7%), respectively, while heart rate (HR) increased by 24.7% (17.0% to 32.9%). Cardiac output (CO) showed no significant variation.

During a prolonged stay of 1 week at HA (from v2 to v3), a trend towards baseline (v1) was evident across various indices (i.e. ba-SBP, DBP, MAP, ca-PP, ba-PP, HR and SEVR), although no significant differences were observed when v3 was compared to v2. Upon returning to SL (from v3 to v4), most indices returned to levels close to the baseline. In particular, PWV and DBP showed significant decreases by 12.3% (6.9% to 17.3%) and 4.9% (0.4% to 9.2%), respectively, compared with baseline. Sensitivity analyses supported the robustness of the haemodynamic changes across altitudes (Table S4).

Changes in hypoxia-related indices (i.e. blood oxygen saturation, red blood cell count, haematocrit and haemoglobin) at different altitudes were consistent with those in haemodynamic indices (Fig. 1b).

We identified 350 genes involved in six pathway categories essential for haemodynamic regulation (Table S5). These pathways included vasoactive substance synthesis, oxidative stress response, vascular smooth muscle cell (VSMC) contraction, cardiac muscle contraction, aldosterone-mediated sodium reabsorption and carbonic anhydrase. Further details are provided in the Supplementary Methods and Table S5.

In general, the expression levels of various genes within these pathways demonstrated significant changes at v2 compared with v1. These changes became even more pronounced at v3. At v4, most gene-expression levels became comparable to the baseline after participants returned to SL (Fig. 1c).

We further analysed selected key genes with essential functions in these pathways (Table S5). Overall, gene-expression changes reflected a pattern of adaptive responses to HA exposure. Specifically, genes related to both the activation and counterbalance of haemodynamic effects were simultaneously altered at HA, indicating a concerted effort to maintain homeostasis (Fig. 1d and Table S6). We observed enhanced vasoconstrictor hormone gene expression at HA, marked by the upregulation of genes encoding epinephrine (*PNMT*), renin (*CTSB*), angiotensin II (*CTSG*) and endothelin-1 (*EDN1*). This was counterbalanced by the increased expressions of genes encoding vasodilators (atrial natriuretic peptide: *NPPA*; prostaglandin D2: *PTGDS*) and vasodilator receptors (prostaglandin I2 receptor: *PTGIR*; calcitonin gene-related peptide receptors: *RAMP1* and *RAMP3*).

The adaptive responses of gene expression were also evidenced on the downstream pathways of the vasoactive hormones (Fig. 1d and Table S6). At HA, the expressions of genes responsible for the contraction of VSMCs (calmodulin: *CALM2* and *CALM3*; myosin light chain: *MYL6, MYL6B* and *MYL9*; actin: *ACTA2*; caldesmon: *CALD1*) and cardiac muscle cells (myosin light chain: *MYL4*; tropomyosin: *TPM1* and *TPM2*; troponin C: *TNNC1*) were upregulated. This was counterbalanced by the downregulation of genes encoding sarcoplasmic reticulum calcium channels in VSMCs (inositol 1,4,5-trisphosphate receptors: *ITPR1, ITPR2* and *ITPR3*) and cardiac muscle cells (cardiac Ca^2+^ ATPase: *ATP2A2*, aspartate beta-hydroxylase: *ASPH*). A similar phenomenon was observed in oxidative stress signalling, with both pro- (NADPH oxidase: *CYBA* and *NCF1*) and antioxidant enzymes (superoxide dismutase 1: *SOD1*; glutathione peroxidase: *GPX1* and *GPX4*; thioredoxin: *TXN* and *TXN2*) being stimulated at HA. The adaptive response also manifested as the expression of different members within a gene family (i.e. a set of genes with similar sequences and functions) being oppositely regulated at HA (e.g. calmodulin: *CALM1, CALM2* and *CALM3*; tropomyosin: *TPM1, TPM2* and *TPM3*; glutathione peroxidase: *GPX1, GPX3, GPX4* and *GPX7*; superoxide dismutase: *SOD1* and *SOD2*).

At HA, an activation of the carbonic anhydrase pathway was also noted, evidenced by elevated expressions of carbonic anhydrase (*CA1* and *CA2*), anion exchange protein 1 (*SLC4A1*), aquaporin 1 (*AQP1*) and Rh-associated glycoprotein (*RHAG*). Conversely, we observed a suppression of the aldosterone-mediated sodium reabsorption pathway, characterized by decreased expression of aldosterone receptor (*NR3C2*) and increased expression of E3 ubiquitin-protein ligase (*NEDD4L*) (Fig. 1d and Table S6). Genes that were not differentially expressed at HA are shown in Fig. S1.

The adaptive responses of gene expression for either the activation or counterbalance of haemodynamic effects were generally more pronounced following prolonged HA exposure (v3 vs. v2) (Table S6). Notably, HA-induced changes in gene expression were largely reversible upon returning to SL, as most genes were not differentially expressed at v4 compared to v1. We also observed that, compared with the baseline (v1), most genes involved in the carbonic anhydrase pathway (*CA1, AQP1* and *SLC4A1*) were upregulated at v2 and v3 and were downregulated at v4.

Significant correlations were observed between the expressions of various genes and haemodynamic indices (Fig. S2), further corroborating the regulatory roles of these pathways in haemodynamics.

We observed significant increases in DBP and MAP but not SBP from SL to HA, suggesting that HA exposure mainly induced acute peripheral resistance, while arterial stiffness was affected to a lesser extent [8], as also indicated by the nonsignificant change in PWV. We observed a significant decrease in SV, i.e. the volume of blood pumped per beat, implying an elevated risk for myocardial ischaemia. This risk was further supported by the decreased SEVR—an indicator of the ratio of myocardial oxygen supply to demand [9].

Peripheral and central haemodynamic indices generally stabilized with prolonged HA exposure, with some even returning to baseline levels. This is a sign of acclimatization to the HA environment. Additionally, the haemodynamic changes aligned with those of hypoxia-related indices, reinforcing the rationality of the acclimatization. The haemodynamic indices largely returned to baseline levels after returning to SL, indicating the reversible nature of HA-induced haemodynamic changes. To the best of our knowledge, the current study is the first to report significant PWV and DBP decreases among healthy young adults after returning from HA to SL, suggesting a health benefit of a reduction in arterial stiffness and peripheral vascular resistance. A previous study has also reported the benefits of intermittent hypoxia training on cardiovascular health, as indicated by reduced peripheral BP and HR along with increased endothelial nitric oxide production [10]. Our findings imply that a short period (within 3 weeks) of HA exposure may be favourable for cardiovascular health, warranting further investigation to validate this hypothesis.

We found a complex array of transcriptional changes underlying the haemodynamic responses to HA exposure, with both the activation and counterbalance of haemodynamic pathways observed at HA. Upon arrival at HA, we observed upregulation of genes encoding several vasoconstrictive hormones involved in the sympathetic nervous system, renin–angiotensin system and endothelial function. Meanwhile, genes encoding vasodilators and vasodilator receptors were also upregulated. Additionally, downstream pathways of these hormones involving the contraction and dilatation of VSMCs and cardiac muscle cells were concomitantly stimulated at HA. We also observed the upregulation of genes encoding pro- and antioxidant enzymes at HA. The adaptive regulation was also evidenced by an activated carbonic anhydrase pathway and suppressed aldosterone signalling. These physiological responses may create homeostasis, allowing the body to acclimatize to HA [3]. Notably, changes in the expression of several genes were more pronounced after an extended HA stay (∼1 week), indicating enhanced physiological responses.

After participants returned to SL, we observed a remarkable genetic reset, aligning with the haemodynamic changes and illustrating the body's capacity to recover from short-term physiological stress at HA. Notably, we found that several genes involved in the carbonic anhydrase pathway that were upregulated at HA became downregulated after returning to SL, which might have contributed to the decreased DBP and PWV observed after returning to SL. Leveraging transcriptomic analysis, our work extends beyond limited biomarkers and comprehensively elucidates the mechanisms of haemodynamic acclimatization to HA and its reversibility after descending to SL.

The sample size of this study is somewhat small, limiting the generalizability of the findings. Additionally, peripheral blood transcriptome analysis may not fully reflect haemodynamic changes in target organs. Finally, certain transcripts involved in the pathways were not detected due to the limited sensitivity of the transcriptomic analysis. More sensitive methods are required to validate our findings in further studies.

In conclusion, this study illustrated the acclimatization and reversibility of the haemodynamic effects of HA exposure in healthy adults. These effects were regulated by the activation and counterbalance of pathways involved in the synthesis of vasoactive substances, vascular and cardiac muscle contraction, oxidative stress, sodium reabsorption and carbonic anhydrase activity. Exploring interventions that target these pathways holds promise for protecting the health of individuals travelling to HA.

## Supplementary Material

nwaf203_Supplemental_File

## Data Availability

The code used in this study can be found in the Supplementary Materials. Data are available from the corresponding authors on reasonable request.

## References

[1] Cornwell WK III, Baggish AL, Bhatta YK et al. J Am Heart Assoc 2021; 10: e023225. 10.1161/JAHA.121.023225 34496612 PMC8649141

[2] Xu J, Xu Y, Hu L et al. Acta Geographica Sinica 2020; 75: 1406–17.

[3] Richalet JP, Hermand E, Lhuissier FJ. Nat Rev Cardiol 2023; 21: 1–14. 10.1038/s41569-023-00924-9 37783743

[4] Berger MM, Sareban M, Bärtsch P. J Appl Physiol 2020; 128: 952–9. 10.1152/japplphysiol.00305.2019 31829805

[5] Luks AM, Hackett PH. N Engl J Med 2022; 386: 364–73. 10.1056/NEJMra2104829 35081281

[6] He B, Wang J, Qian G et al. PLoS One 2013; 8: e62072. 10.1371/journal.pone.0062072 23650508 PMC3641122

[7] Narvaez-Guerra O, Herrera-Enriquez K, Medina-Lezama J et al. Hypertension 2018; 72: 567–78. 10.1161/HYPERTENSIONAHA.118.11140 30354760 PMC6205208

[8] McEniery CM, Cockcroft JR, Roman MJ et al. Eur Heart J 2014; 35: 1719–25. 10.1093/eurheartj/eht565 24459197 PMC4155427

[9] Salvi P, Revera M, Faini A et al. Hypertension 2013; 61: 793–9. 10.1161/HYPERTENSIONAHA.111.00707 23438935

[10] Muangritdech N, Hamlin MJ, Sawanyawisuth K et al. Eur J Appl Physiol 2020; 120: 1815–26. 10.1007/s00421-020-04410-9 32524226

