# Supplementary material for: Human adaptation to high altitude: acclimatization and reversibility of haemodynamics
Source: Natl Sci Rev. 2025 May 21;12(7):nwaf203. doi: 10.1093/nsr/nwaf203 (PMC12202200; doi:10.1093/nsr/nwaf203)
Supplement: nwaf203_Supplemental_File [file nwaf203_supplemental_file.docx]

**SUPPLEMENTAL MATERIALS**

**Human Adaptation to High Altitude: Acclimatization and Reversibility of Haemodynamics**

**Supplementary Methods.**

***Participants***

We recruited healthy adults living at low altitude (Beijing, 44 m above sea level [ASL]), who had no known cardiovascular diseases, were not on chronic cardiovascular therapy, had no history of severe mountain sickness and had not been exposed to altitudes >2500 m in the 2 months prior to the study. All participants underwent a comprehensive medical examination to assess fitness for the expedition before enrolment. Baseline data on age, sex, body mass index, occupation, smoking status, education, income, diet and other living habits were recorded. Following a previous study [1] that had a similar design, we have estimated by paired *t*-test (two sided) that 25 participants were sufficient to demonstrate with *p*<0.05 and a power of 0.8 for the differences in diastolic blood pressure (DBP) and pulse wave velocity (PWV) between sea level and high altitude. Accordingly, with a power of 0.8, the estimated sample size was 23 for brachial artery systolic blood pressure (ba-SBP), 21 for central aortic systolic blood pressure (ca-SBP) and mean arterial pressure (MAP), 14 for subendocardial viability ratio (SEVR), 14 for heart rate (HR), 19 for haemoglobin (HGB), and 5 for blood oxygen saturation (SpO_2_). Finally, 21 participants were recruited as a compromise between participant availability and the estimated sample size. This study was approved by the Institutional Review Board of the Health Science Center of Peking University (approval no. IRB00001052-19062), and written informed consent was obtained from all participants.

***Study procedures***

The study timeline is illustrated in Fig. 1a. After recruitment, participants completed the initial clinical visit (v1) in Beijing, and in the following week, travelled by air to Lhasa (elevation: 3650 m ASL), where they spent a week. Subsequently, they proceeded by car to the Mt. Qomolangma region (elevation: 4300−5200 m ASL), where they resided and worked. The second visit (v2) took place within 5 days upon arrival at the region, and the third visit (v3) occurred after a 7-day interval. Of the 21 participants, 7 were living and working at 5200 m ASL (Mt. Qomolangma base camp), while 14 resided at 4300 m ASL (Zhaxizong village) and commuted to work at 5200 m ASL during the daytime. The participants returned to Beijing within a week after the third visit. The final visit (v4) was conducted within 3.5 months of the participants’ return, and most (n = 19/21) completed the visit within 2 months. All clinical visits were performed in the morning (8:00−9:30 AM) either in clinic rooms in Beijing or tents at 5200 m ASL (n = 7), or rooms at 4300 m ASL (n = 14). Participants were instructed to fast overnight and abstain from smoking or strenuous physical activity for 1 day prior to each clinical visit. During each visit, haemodynamic indices and SpO_2_ were measured. Blood samples were collected for complete blood count and transcriptome sequencing. A follow-up questionnaire was administered to collect information on oxygen and altitude sickness medication use, as well as any symptoms (headache, gastrointestinal symptoms, fatigue and dizziness) experienced during the previous 3 days.

***Health measurements***

*Hypoxia-related indices*

Plasma red blood cell (RBC) counts, HGB and haematocrit (HCT) were analysed using biochemical analysers (Beijing: SYSMEX-XN-1000, Sysmex Corp.; Mt. Qomolangma region: BC-3000Plus, Shenzhen Mindray Bio-Medical Electronics Co., Ltd.) within 3 h after sample collection with EDTA-coated tubes. SpO_2_ level was assessed using a pulse oximeter (Oxiband Pulse Oximeter, Shenzhen Viatom Technology Co., Ltd) with an accuracy of ≥98% and a resolution of 1%.

*Haemodynamic indices*

The Vicorder device (80 Beats Medical, Berlin, Germany) was used to measure haemodynamic indices, following the manufacturer’s instructions. Participants assumed a semi-supine position with their head and shoulders elevated by approximately 30° and remained still. At the base camp, an electric heated blanket was used to maintain a comfortable temperature. A cuff was placed on the upper right arm for the measurement of brachial artery blood pressure parameters, including ba-SBP, DBP, MAP, brachial artery pulse pressure (ba-PP) and pulse wave analysis. DBP and MAP remain largely unchanged throughout the large conduit arteries [2]; therefore, only peripheral DBP and MAP levels were measured as representatives. Central aortic waveforms were calculated from brachial pulse waveforms using a mathematical function. The Vicorder device automatically generated central haemodynamic parameters, i.e., ca-SBP and central aortic pulse pressure (ca-PP), as well as cardiac function indicators, including HR, stroke volume (SV), cardiac output (CO) and SEVR. Brachial-ankle PWV, an indicator of arterial stiffness, was assessed using two pressure cuffs, one placed at the brachial and another at the ankle point to detect the pulse. The distance between the centres of the two cuffs was measured using a tape and fed into the Vicorder system for PWV calculation [3]. All measurements were performed three times, and averages were used for further analyses.

*Transcriptomic analysis*

Peripheral blood provides valuable insights into physiopathologic changes in the body, as it constantly interacts with all tissues and responds to macro- and micro-environmental changes [4, 5]. This notion is supported by previous findings that gene expression in peripheral blood cells is significantly correlated with those in the heart [6] and central nervous system [7]. Additionally, several studies have demonstrated that peripheral blood transcripts can serve as biomarkers of environmental exposure [8] and cardiovascular disease progression where haemodynamics are closely involved, such as coronary artery disease [9], atherosclerosis [10], hypertension [11] and heart failure [12]. Therefore, peripheral blood transcriptomic analysis was used to investigate HA-induced haemodynamic effects.

Blood samples for RNA extraction were collected using Paxgene Blood RNA tubes (PreAnalytiX, QIAGEN, Germany). The tubes were left at room temperature for homogenisation for 2 h, followed by 24 h storage in a –20°C freezer, and then they were stored in dry ice (at Mt. Qomolangma base camp and during transfer) or a –80°C freezer (after transfer to Beijing) until further analysis.

RNA extraction was performed using the PAXgene Blood RNA Kit (PreAnalytiX, QIAGEN, Germany) following the manufacturer’s protocol. The concentration and purity of extracted RNA were measured using a NanoDrop spectrophotometer (Thermo Fisher Scientific, Waltham, MA, USA). The RNA integrity number, measured using an Agilent 5400 Bioanalyzer (Agilent Technologies, Inc., Santa Clara, CA, USA), was 7.87 ± 1.43 in this study, indicating high RNA integrity.

Transcriptome sequencing was conducted using the Illumina NovaSeq system (San Diego, CA, USA). After mapping with the Ensembl reference genome [13], an indicator of relative expression levels of RNA, namely, fragments per kilobase of transcript per million mapped reads [14], were calculated for subsequent statistical analysis.

***Statistical analyses***

Descriptive statistics were calculated for demographic characteristics and health indices. All indices were natural log-transformed due to their right-skewed distributions. Given the repeated-measurements design, we used linear mixed-effect (LME) models to estimate the associations between health indices and altitude (incorporated as a categorical variable indicating four clinical visits), with a random participant-specific intercept used to control for within-participant variation among repeated measurements. The LME models were adjusted for age, sex and body mass index. All estimates are reported as percentage differences with 95% confidence intervals (CIs) compared to baseline (v1). We also examined potential differences between the effects upon arrival (v2) and after a period of stay at HA (v3), calculated as: ($\hat{Q}_{1}-\hat{Q}_{2}$) ± 1.96$\sqrt{{\hat{{SE}_{1}}}^{2}+{\hat{{SE}_{2}}}^{2}}$, where $\hat{Q}_{1}$ and $\hat{Q}_{2}$ are regression coefficients, and $\hat{{SE}_{1}}$ and $\hat{{SE}_{2}}$ are standard errors. Statistical significance was defined as *p* < 0.05. The *p*-values were adjusted for multiple comparisons using the Benjamini-Hochberg method with an overall false discovery rate of 5%.

To elucidate the mechanisms underlying altitude-induced haemodynamic effects, we conducted transcriptomic analysis using the LME models to investigate altitude-related changes in gene expression levels. Transcripts with a detection rate > 50% qualified for statistical analyses. As the study focused on haemodynamics, an analytical approach targeting critical biological pathways involved in haemodynamic modulations was used, including blood pressure, arterial stiffness and cardiac function. We searched the literature for general pathophysiologic pathways underlying haemodynamics, as well as those suggested to mediate the haemodynamic effects of HA, and referred to the pathways and involved genes in well-established databases, including Kyoto Encyclopedia of Genes and Genomes, WikiPathways and Reactome. We identified six major pathway categories, including vasoactive substance synthesis, oxidative stress response, vascular smooth muscle cell (VSMC) contraction, cardiac muscle contraction, aldosterone-mediated sodium reabsorption and carbonic anhydrase. To investigate the key biological processes that mediate HA-induced haemodynamic changes, we focused on genes with essential roles in the signalling cascades of the pathways, including the synthesis of signalling molecules (e.g., hormones), receptors and ion channels, and the downstream functional proteins (Supplementary Table S5) [15].

To ensure the reliability of the LME model results, we further conducted sensitivity analyses including additional adjustment for smoking status (current smokers vs. never smokers) as well as for altitude sickness medication and oxygen use (yes vs. no) which could have impacted the haemodynamics, and HR and MAP adjustment while estimating the effect of HA on PWV, in consideration of the potential influence of HR and BP on arterial stiffness [1]. All analyses were conducted using the lme4 package in R statistical software (version 3.5.3).

**Supplemental Materials References**

1 Revera, M. *et al.* Renin–angiotensin–aldosterone system is not involved in the arterial stiffening induced by acute and prolonged exposure to high altitude. *Hypertension* **70**, 75-84 (2017).

2 Pauca, A. L., Wallenhaupt, S. L., Kon, N. D. & Tucker, W. Y. Does radial artery pressure accurately reflect aortic pressure? *Chest* **102**, 1193-1198 (1992).

3 Teren, A., Beutner, F., Wirkner, K., Löffler, M. & Scholz, M. Relationship between determinants of arterial stiffness assessed by diastolic and suprasystolic pulse oscillometry: comparison of vicorder and vascular explorer. *Medicine* **95**, e2963 (2016).

4 Liew, C.-C., Ma, J., Tang, H.-C., Zheng, R. & Dempsey, A. A. The peripheral blood transcriptome dynamically reflects system wide biology: a potential diagnostic tool. *J Lab Clin Med* **147**, 126-132 (2006).

5 Mohr, S. & Liew, C.-C. The peripheral-blood transcriptome: new insights into disease and risk assessment. *Trends Mol Med* **13**, 422-432 (2007).

6 Gerling, I. C. *et al.* Gene expression profiles of peripheral blood mononuclear cells reveal transcriptional signatures as novel biomarkers of cardiac remodeling in rats with aldosteronism and hypertensive heart disease. *JACC Heart Fail* **1**, 469-476 (2013).

7 Sullivan, P. F., Fan, C. & Perou, C. M. Evaluating the comparability of gene expression in blood and brain. *Am J Med Genet B Neuropsychiatr* **141**, 261-268 (2006).

8 Bushel, P. *et al.* Blood gene expression signatures predict exposure levels. *Proc Natl Acad Sci U. S. A.* **104**, 18211-18216 (2007).

9 Ma, J. & Liew, C.-C. Gene profiling identifies secreted protein transcripts from peripheral blood cells in coronary artery disease. *J Mol Cell Cardiol* **35**, 993-998 (2003).

10 Patino, W. D. *et al.* Circulating transcriptome reveals markers of atherosclerosis. *Proc Natl Acad Sci U. S. A.* **102**, 3423-3428 (2005).

11 Chon, H. *et al.* Broadly altered gene expression in blood leukocytes in essential hypertension is absent during treatment. *Hypertension* **43**, 947-951 (2004).

12 Deng, M. C. A peripheral blood transcriptome biomarker test to diagnose functional recovery potential in advanced heart failure. *Biomark Med* **12**, 619-635 (2018).

13 Cunningham, F. *et al.* Ensembl 2022. *Nucleic Acids Res.* **50**, D988-D995 (2022).

14 Trapnell, C. *et al.* Transcript assembly and quantification by RNA-Seq reveals unannotated transcripts and isoform switching during cell differentiation. *Nat Biotechnol* **28**, 511-515 (2010).

15 Brivanlou, A. H. & Darnell Jr, J. E. Signal transduction and the control of gene expression. *Science* **295**, 813-818 (2002).

16 William Tank, A. & Lee Wong, D. Peripheral and central effects of circulating catecholamines. *Compr Physiol* **5**, 1-15 (2011).

17 Holwerda, S. W. *et al.* Elevated muscle sympathetic nerve activity contributes to central artery stiffness in young and middle-age/older adults. *Hypertension* **73**, 1025-1035 (2019).

18 Mazzeo, R. S. *et al.* Catecholamine response during 12 days of high-altitude exposure (4,300 m) in women. *J Appl Physiol* **84**, 1151-1157 (1998).

19 Parati, G. *et al.* Changes in 24 h ambulatory blood pressure and effects of angiotensin II receptor blockade during acute and prolonged high-altitude exposure: a randomized clinical trial. *Eur Heart J* **35**, 3113-3122 (2014).

20 Paz Ocaranza, M. *et al.* Counter-regulatory renin–angiotensin system in cardiovascular disease. *Nat Rev Cardiol* **17**, 116-129 (2020).

21 Zieman, S. J., Melenovsky, V. & Kass, D. A. Mechanisms, pathophysiology, and therapy of arterial stiffness. *Arterioscler Thromb Vasc Biol* **25**, 932-943 (2005).

22 Kostov, K. The causal relationship between endothelin-1 and hypertension: focusing on endothelial dysfunction, arterial stiffness, vascular remodeling, and blood pressure regulation. *Life* **11**, 986 (2021).

23 Modesti, P. A. *et al.* Role of endothelin-1 in exposure to high altitude: Acute Mountain Sickness and Endothelin-1 (ACME-1) study. *Circulation* **114**, 1410-1416 (2006).

24 Farah, C., Michel, L. Y. & Balligand, J.-L. Nitric oxide signalling in cardiovascular health and disease. *Nat Rev Cardiol* **15**, 292-316 (2018).

25 Wilkinson, I. B., Franklin, S. S. & Cockcroft, J. R. Nitric oxide and the regulation of large artery stiffness: from physiology to pharmacology. *Hypertension* **44**, 112-116 (2004).

26 Beall, C. M., Laskowski, D. & Erzurum, S. C. Nitric oxide in adaptation to altitude. *Free Radic Biol Med* **52**, 1123-1134 (2012).

27 Daniels, L. B. & Maisel, A. S. Natriuretic peptides. *J Am Coll Cardiol* **50**, 2357-2368 (2007).

28 Bálint, L., Nelson-Maney, N. P., Tian, Y., Serafin, S. D. & Caron, K. M. Clinical potential of adrenomedullin signaling in the cardiovascular system. *Circ Res* **132**, 1185-1202 (2023).

29 Goldfarb-Rumyantzev, A. S. & Alper, S. L. Short-term responses of the kidney to high altitude in mountain climbers. *Nephrol Dial Transplant* **29**, 497-506 (2014).

30 Bailey, D. M. *et al.* Transcerebral exchange kinetics of nitrite and calcitonin gene-related peptide in acute mountain sickness: evidence against trigeminovascular activation? *Stroke* **40**, 2205-2208 (2009).

31 Hasbak, P., Lundby, C., Olsen, N. V., Schifter, S. & Kanstrup, I.-L. Calcitonin gene-related peptide and adrenomedullin release in humans: effects of exercise and hypoxia. *Regul Pept* **108**, 89-95 (2002).

32 Imig, J. D. Eicosanoid blood vessel regulation in physiological and pathological states. *Clin Sci* **134**, 2707-2727 (2020).

33 Burtscher, M., Likar, R., Nachbauer, W. & Philadelphy, M. Aspirin for prophylaxis against headache at high altitudes: randomised, double blind, placebo controlled trial. *Br Med J* **316**, 1057 (1998).

34 Mrakic-Sposta, S. *et al.* OxInflammation at high altitudes: A proof of concept from the Himalayas. *Antioxidants* **11**, 368 (2022).

35 Touyz, R. M. *et al.* Vascular smooth muscle contraction in hypertension. *Cardiovasc Res* **114**, 529-539 (2018).

36 Lacolley, P., Regnault, V., Segers, P. & Laurent, S. Vascular smooth muscle cells and arterial stiffening: relevance in development, aging, and disease. *Physiol Rev* **97**, 1555-1617 (2017).

37 Narvaez-Guerra, O., Herrera-Enriquez, K., Medina-Lezama, J. & Chirinos, J. A. Systemic hypertension at high altitude. *Hypertension* **72**, 567-578 (2018).

38 Ripa, R., George, T. & Sattar, Y. Physiology, cardiac muscle. (2021).

39 Davis, J. P. & Tikunova, S. B. Ca^2+^ exchange with troponin C and cardiac muscle dynamics. *Cardiovasc Res* **77**, 619-626 (2008).

40 Klausen, K. Cardiac output in man in rest and work during and after acclimatization to 3,800 m. *J Appl Physiol* **21**, 609-616 (1966).

41 Dubois-Deruy, E., Peugnet, V., Turkieh, A. & Pinet, F. Oxidative stress in cardiovascular diseases. *Antioxidants* **9**, 864 (2020).

42 Pena, E., El Alam, S., Siques, P. & Brito, J. Oxidative stress and diseases associated with high-altitude exposure. *Antioxidants* **11**, 267 (2022).

43 Briet, M. & Schiffrin, E. L. Aldosterone: effects on the kidney and cardiovascular system. *Nat Rev Nephrol* **6**, 261-273 (2010).

44 Rajkumar, P. & Pluznick, J. L. Acid-base regulation in the renal proximal tubules: using novel pH sensors to maintain homeostasis. *Am J Physiol Renal Physiol* **315**, F1187-F1190 (2018).

45 Swenson, E. R. New insights into carbonic anhydrase inhibition, vasodilation, and treatment of hypertensive-related diseases. *Curr Hypertens Rep* **16**, 1-11 (2014).

46 Salvi, P. *et al.* Changes in subendocardial viability ratio with acute high-altitude exposure and protective role of acetazolamide. *Hypertension* **61**, 793-799 (2013).

47 Parati, G. *et al.* Effects of acetazolamide on central blood pressure, peripheral blood pressure, and arterial distensibility at acute high altitude exposure. *Eur Heart J* **34**, 759-766 (2013).

**Supplementary Tables.**

Table S1. Characteristics of the study participants.

Table S2. Levels of health indices at four clinic visits.

Table S3. Percent changes (means and 95% confidence intervals) in haemodynamic and hypoxia-related indices at different altitudes.

Table S4. Sensitivity analyses for the effects of high altitude exposure on the selected haemodynamic indices.

Table S5. Selected pathways that potentially mediate the haemodynamic effects posed by high altitude exposure.

Table S6. Adaptive responses (activation vs. counterbalance) of the haemodynamics-regulating pathways to high altitude exposure.

**Supplementary Figures.**

Fig. S1. Changes in the key genes at four clinic visits.

Fig. S2. Correlations between haemodynamic indices and key genes involved in haemodynamics-regulating pathways.

**Statistical Analysis Code**

**Table S1. Characteristics of the study participants.**

| Characteristics | N (%) / Mean ± SD |
| --- | --- |
| No. of participants | 21 (100) |
| No. of clinic visits ^a^ | 83 (100) |
| *Demographic characteristics* |  |
| Age (year) | 32.5 ± 10.1 |
| Body mass index (kg/m^2^) | 23.0 ± 3.0 |
| Sex |  |
| Male | 13 (62) |
| Female | 8 (38) |
| Smoking status |  |
| Never smokers | 16 (76) |
| Current smokers | 5 (24) |
| Education level |  |
| University | 21 (100) |
| Below university | 0 (0) |
| Birthplace altitude (m) |  |
| <1500 | 17 (81) |
| 1500–2500 | 4 (19) |
| ≥2500 | 0 (0) |
| *Symptoms at high altitude* |  |
| Headache |  |
| Absent | 5 (24) |
| Mild | 8 (38) |
| Moderate | 7 (33) |
| Severe | 1 (5) |
| Gastrointestinal symptoms |  |
| Absent | 9 (43) |
| Mild | 10 (48) |
| Moderate | 2 (9) |
| Severe | 0 (0) |
| Fatigue |  |
| Absent | 4 (19) |
| Mild | 9 (43) |
| Moderate | 8 (38) |
| Severe | 0 (0) |
| Dizziness |  |
| Absent | 8 (38) |
| Mild | 9 (43) |
| Moderate | 4 (19) |
| Severe | 0 (0) |
| *Altitude sickness treatment* |  |
| Oxygen therapy |  |
| Yes | 10 (48) |
| No | 11 (52) |
| Altitude sickness medication |  |
| Yes | 3 (14) |
| No | 18 (86) |

^a^ 21, 21, 20, and 21 participants attended the first, the second, the third, and the final clinical visit, respectively.

Abbreviations: SD, standard deviation.

**Table S2. Levels of health indices at four clinic visits.**

| Category | Health Indices | Unit | v1  (44 m) | v2  (4300−5200 m) | v3  (4300−5200 m) | v4  (44 m) |
| --- | --- | --- | --- | --- | --- | --- |
| Blood pressure | ca-SBP | mmHg | 121.9 ± 7.9 | 123.9 ± 9.5 | 124.6 ± 8.1 | 120.5 ± 9.1 |
|  | ba-SBP | mmHg | 126.8 ± 8.1 | 131.4 ± 8.8 | 130.2 ± 8.3 | 123.3 ± 8.7 |
|  | DBP | mmHg | 67.9 ± 6.2 | 76.0 ± 7.0 | 74.5 ± 6.5 | 65.1 ± 7.6 |
|  | MAP | mmHg | 93.5 ± 6.7 | 99.4 ± 8.0 | 98.9 ± 7.0 | 91.3 ± 7.9 |
|  | ca-PP | mmHg | 54.0 ± 6.2 | 47.8 ± 5.3 | 50.1 ± 4.3 | 55.4 ± 6.3 |
|  | ba-PP | mmHg | 58.9 ± 5.5 | 55.3 ± 5.7 | 55.7 ± 5.2 | 58.2 ± 5.8 |
| Arterial stiffness | PWV | m/s | 10.2 ± 1.2 | 10.6 ± 1.4 | 10.7 ± 1.2 | 9.1 ± 1.3 |
| Cardiac function | HR | BPM | 68.3 ± 6.2 | 86.4 ± 12.5 | 82.0 ± 9.2 | 66.6 ± 9.8 |
|  | SV | mL | 112.3 ± 14.1 | 92.9 ± 9.8 | 92.3 ± 9.7 | 110.7 ± 12.1 |
|  | CO | L/min | 7.7 ± 1.1 | 8.0 ± 1.4 | 7.5 ± 1.0 | 7.4 ± 1.3 |
|  | SEVR | - | 1.5 ± 0.2 | 1.2 ± 0.2 | 1.4 ± 0.2 | 1.5 ± 0.2 |
| Hypoxia | SpO_2_ | % | 96.7 ± 1.2 | 84.9 ± 3.8 | 86.6 ± 3.1 | 96.0 ± 2.6 |
|  | RBC | 10^12^/L | 4.9 ± 0.5 | 5.5 ± 0.4 | 5.5 ± 0.5 | 5.1 ± 0.6 |
|  | HCT | % | 45.6 ± 3.9 | 52.8 ± 4.7 | 53.0 ± 4.6 | 46.0 ± 4.4 |
|  | HGB | g/L | 150.5 ± 15.3 | 169.7 ± 15.0 | 169.5 ± 14.9 | 152.8 ± 16.1 |

Abbreviations: ca-SBP, central aortic systolic blood pressure; ba-SBP, brachial artery systolic blood pressure; DBP, diastolic blood pressure; MAP, mean arterial pressure; ca-PP, central aortic pulse pressure; ba-PP, brachial artery pulse pressure; PWV, pulse wave velocity; HR, heart rate; SV, stroke volume; CO, cardiac output; SEVR, subendocardial viability ratio; SpO_2_, blood oxygen saturation; RBC, red blood cell; HCT, haematocrit; HGB, haemoglobin.

Data are expressed as mean ± SD (standard deviation).

**Table S3. Percent changes (means and 95% confidence intervals) in haemodynamic and hypoxia-related indices at different altitudes.**

| Health indices | Visit | Estimate (95% CI) ^a^ | *p*-value | FDR |
| --- | --- | --- | --- | --- |
| ca-SBP | v2 | 1.0 (-2.5, 4.7) | 0.574 | 0.574 |
|  | v3 | 1.8 (-1.8, 5.6) | 0.336 | 0.360 |
|  | v4 | -1.6 (-5.1, 2.1) | 0.390 | 0.756 |
| ba-SBP | v2 | 3.1 (-0.2, 6.5) | 0.070 | 0.088 |
|  | v3 | 2.3 (-1.1, 5.8) | 0.184 | 0.213 |
|  | v4 | -3.1 (-6.3, 0.2) | 0.068 | 0.342 |
| DBP | v2 | 11.0 (6.1, 16.1) | < 0.001 | < 0.001 |
|  | v3 | 9.1 (4.2, 14.2) | < 0.001 | < 0.001 |
|  | v4 | -4.9 (-9.2, -0.4) | 0.033 | 0.249 |
| MAP | v2 | 5.6 (1.8, 9.6) | 0.004 | 0.005 |
|  | v3 | 5.3 (1.5, 9.3) | 0.006 | 0.012 |
|  | v4 | -2.9 (-6.5, 0.8) | 0.126 | 0.472 |
| ca-PP | v2 | -11.4 (-16.9, -5.7) | < 0.001 | < 0.001 |
|  | v3 | -7.2 (-13.0, -1.0) | 0.022 | 0.037 |
|  | v4 | 2.3 (-4.1, 9.1) | 0.491 | 0.819 |
| ba-PP | v2 | -6.2 (-11.3, -0.8) | 0.025 | 0.034 |
|  | v3 | -5.6 (-10.8, -0.1) | 0.047 | 0.070 |
|  | v4 | -1.3 (-6.8, 4.5) | 0.646 | 0.881 |
| PWV | v2 | 3.4 (-2.4, 9.6) | 0.255 | 0.295 |
|  | v3 | 4.7 (-1.3, 11.0) | 0.128 | 0.175 |
|  | v4 | -12.3 (-17.3, -6.9) | < 0.001 | < 0.001 |
| HR | v2 | 24.7 (17.0, 32.9) | < 0.001 | < 0.001 |
|  | v3 | 18.3 (10.9, 26.2) | < 0.001 | < 0.001 |
|  | v4 | -3.5 (-9.6, 3.0) | 0.288 | 0.719 |
| SV | v2 | -17.2 (-22.6, -11.5) | < 0.001 | < 0.001 |
|  | v3 | -17.4 (-22.9, -11.6) | < 0.001 | < 0.001 |
|  | v4 | -0.7 (-7.3, 6.3) | 0.832 | 0.960 |
| CO | v2 | 3.8 (-5.4, 13.8) | 0.432 | 0.463 |
|  | v3 | -1.8 (-10.5, 7.8) | 0.706 | 0.706 |
|  | v4 | -3.9 (-12.6, 5.6) | 0.403 | 0.756 |
| SEVR | v2 | -18.6 (-25.7, -10.8) | < 0.001 | < 0.001 |
|  | v3 | -6.5 (-14.8, 2.5) | 0.152 | 0.190 |
|  | v4 | -0.4 (-9.2, 9.4) | 0.938 | 0.966 |
| SpO_2_ | v2 | -11.9 (-14.2, -9.5) | < 0.001 | < 0.001 |
|  | v3 | -10.3 (-12.6, -8.0) | < 0.001 | < 0.001 |
|  | v4 | -0.7 (-3.2, 1.8) | 0.574 | 0.861 |
| RBC | v2 | 10.5 (7.2, 13.9) | < 0.001 | < 0.001 |
|  | v3 | 11.4 (8.1, 14.8) | < 0.001 | < 0.001 |
|  | v4 | 1.7 (-1.2, 4.7) | 0.268 | 0.719 |
| HCT | v2 | 14.4 (11.5, 17.4) | < 0.001 | < 0.001 |
|  | v3 | 15.5 (12.5, 18.6) | < 0.001 | < 0.001 |
|  | v4 | -0.4 (-2.9, 2.1) | 0.726965931 | 0.909 |
| HGB | v2 | 11.4 (8.5, 14.4) | < 0.001 | < 0.001 |
|  | v3 | 12.2 (9.3, 15.2) | < 0.001 | < 0.001 |
|  | v4 | 0.1 (-2.4, 2.6) | 0.965 | 0.966 |

^a^ Shown as percent changes in the levels of health indices compared to the first (baseline) clinical visit.

Abbreviations: ca-SBP, central aortic systolic blood pressure; ba-SBP, brachial artery systolic blood pressure; DBP, diastolic blood pressure; MAP, mean arterial pressure; ca-PP, central aortic pulse pressure; ba-PP, brachial artery pulse pressure; PWV, pulse wave velocity; HR, heart rate; SV, stroke volume; CO, cardiac output; SEVR, subendocardial viability ratio; SpO2, blood oxygen saturation; RBC, red blood cell; HCT, haematocrit; HGB, haemoglobin.

**Table S4. Sensitivity analyses for the effects of high altitude exposure on the selected haemodynamic indices.**

| Health indices | Visit | Model 1 | | Model 2 | | Model 3 | | Model 4 | | Model 5 | | Model 6 (only for PWV) | | Model 7 (only for PWV) | |
| --- | --- | --- | --- | --- | --- | --- | --- | --- | --- | --- | --- | --- | --- | --- | --- |
|  |  | Estimate (95% CI) ^a^ | *p*-value | Estimate (95% CI) ^a^ | *p*-value | Estimate (95% CI) ^a^ | *p*-value | Estimate (95% CI) ^a^ | *p*-value | Estimate (95% CI) ^a^ | *p*-value | Estimate (95% CI) ^a^ | *p*-value | Estimate (95% CI) ^a^ | *p*-value |
| DBP | v2 | 11.0 (6.1, 16.1) | < 0.001 | 11.0 (6.1, 16.1) | < 0.001 | 11.6 (6.6, 16.8) | < 0.001 | 11.0 (6.1, 16.1) | < 0.001 | 11.0 (6.1, 16.1) | < 0.001 | - | - | - | - |
|  | v3 | 9.1 (4.2, 14.2) | < 0.001 | 9.2 (4.3, 14.3) | < 0.001 | 9.5 (4.6, 14.6) | < 0.001 | 8.7 (3.8, 13.8) | < 0.001 | 9.1 (4.2, 14.2) | < 0.001 | - | - | - | - |
|  | v4 | -4.9 (-9.2, -0.4) | 0.033 | -4.9 (-9.2, -0.4) | 0.032 | -5.0 (-9.2, -0.5) | 0.029 | -5.3 (-9.6, -0.8) | 0.022 | -4.9 (-9.2, -0.4) | 0.033 | - | - | - | - |
| MAP | v2 | 5.6 (1.8, 9.6) | 0.004 | 5.6 (1.8, 9.5) | 0.004 | 5.8 (1.9, 9.8) | 0.003 | 5.6 (1.8, 9.6) | 0.004 | 5.6 (1.8, 9.6) | 0.004 |  |  |  |  |
|  | v3 | 5.3 (1.5, 9.3) | 0.006 | 5.4 (1.5, 9.4) | 0.006 | 5.4 (1.5, 9.5) | 0.006 | 5.1 (1.2, 9.1) | 0.010 | 5.3 (1.5, 9.3) | 0.006 |  |  |  |  |
|  | v4 | -2.9 (-6.5, 0.8) | 0.126 | -2.9 (-6.5, 0.8) | 0.123 | -2.9 (-6.5, 0.8) | 0.124 | -3.1 (-6.7, 0.6) | 0.103 | -2.9 (-6.5, 0.8) | 0.125 |  |  |  |  |
| ca-PP | v2 | -11.4 (-16.9, -5.7) | < 0.001 | -11.5 (-16.9, -5.7) | < 0.001 | -11.9 (-17.4, -6.0) | < 0.001 | -11.4 (-16.9, -5.6) | < 0.001 | -11.4 (-16.9, -5.7) | <0.001 | - | - | - | - |
|  | v3 | -7.2 (-13.0, -1.0) | 0.022 | -7.1 (-12.9, -1.0) | 0.024 | -7.5 (-13.3, -1.3) | 0.018 | -7.0 (-12.8, -0.8) | 0.029 | -7.2 (-13.0, -1.0) | 0.023 | - | - | - | - |
|  | v4 | 2.3 (-4.1, 9.1) | 0.491 | 2.3 (-4.2, 9.1) | 0.498 | 2.4 (-4.1, 9.2) | 0.482 | 2.5 (-4.0, 9.5) | 0.457 | 2.3 (-4.1, 9.2) | 0.490 | - | - | - | - |
| ba-PP | v2 | -6.2 (-11.3, -0.8) | 0.025 | -6.2 (-11.4, -0.8) | 0.025 | -6.9 (-12.0, -1.4) | 0.014 | -6.2 (-11.3, -0.8) | 0.026 | -6.2 (-11.3, -0.8) | 0.026 | - | - | - | - |
|  | v3 | -5.6 (-10.8, -0.1) | 0.047 | -5.5 (-10.8, 0) | 0.050 | -6.0 (-11.2, -0.5) | 0.032 | -5.4 (-10.7, 0.2) | 0.058 | -5.6 (-10.8, 0) | 0.048 | - | - | - | - |
|  | v4 | -1.3 (-6.8, 4.5) | 0.646 | -1.4 (-6.9, 4.5) | 0.637 | -1.2 (-6.7, 4.5) | 0.663 | -1.1 (-6.7, 4.8) | 0.697 | -1.3 (-6.8, 4.5) | 0.646 | - | - | - | - |
| SV | v2 | -17.2 (-22.6,-11.5) | < 0.001 | -17.2 (-22.6,-11.5) | < 0.001 | -17.6 (-23.1,-11.7) | < 0.001 | -17.2 (-22.6,-11.4) | < 0.001 | -17.3 (-22.7, -11.6) | <0.001 | - | - | - | - |
|  | v3 | -17.4 (-22.9,-11.6) | < 0.001 | -17.4 (-22.9,-11.6) | < 0.001 | -17.7 (-23.1,-11.8) | < 0.001 | -17.2 (-22.8,-11.4) | < 0.001 | -17.5 (-23.0, -11.8) | <0.001 | - | - | - | - |
|  | v4 | -0.7 (-7.3, 6.3) | 0.832 | -0.7 (-7.4, 6.4) | 0.832 | -0.7 (-7.3, 6.4) | 0.838 | -0.5 (-7.2, 6.6) | 0.883 | -0.7 (-7.3, 6.4) | 0.844 | - | - | - | - |
| HR | v2 | 24.7 (17.0, 32.9) | < 0.001 | 24.7 (17.0, 32.9) | < 0.001 | 23.5 (15.8, 31.8) | < 0.001 | 24.7 (17.0, 33.0) | < 0.001 | 24.8 (17.0, 33.0) | <0.001 | - | - | - | - |
|  | v3 | 18.3 (10.9, 26.2) | < 0.001 | 18.2 (10.8, 26.1) | < 0.001 | 17.6 (10.2, 25.5) | < 0.001 | 18.4 (10.9, 26.5) | < 0.001 | 18.4 (10.9, 26.3) | <0.001 | - | - | - | - |
|  | v4 | -3.5 (-9.6, 3.0) | 0.288 | -3.4 (-9.5, 3.0) | 0.290 | -3.4 (-9.4, 3.1) | 0.301 | -3.4 (-9.6, 3.2) | 0.310 | -3.5 (-9.6, 3.0) | 0.284 | - | - | - | - |
| SEVR | v2 | -18.6 (-25.7,-10.8) | < 0.001 | -18.8 (-25.8,-11.0) | < 0.001 | -17.2 (-24.4, -9.2) | < 0.001 | -18.6 (-25.8,-10.8) | < 0.001 | -18.6 (-25.7, -10.8) | <0.001 | - | - | - | - |
|  | v3 | -6.5 (-14.8, 2.5) | 0.152 | -6.5 (-14.8, 2.5) | 0.151 | -5.5 (-13.8, 3.6) | 0.227 | -6.6 (-15.0, 2.6) | 0.153 | -6.5 (-14.8, 2.5) | 0.152 | - | - | - | - |
|  | v4 | -0.4 (-9.2, 9.4) | 0.938 | -0.4 (-9.3, 9.3) | 0.930 | -0.6 (-9.3, 9.0) | 0.901 | -0.4 (-9.4, 9.4) | 0.925 | -0.4 (-9.2, 9.4) | 0.938 | - | - | - | - |
| PWV | v2 | 3.4 (-2.4, 9.6) | 0.255 | 3. 4 (-2.4, 9.6) | 0.261 | 3.6 (-2.4, 10.0) | 0.241 | 3.4 (-2.4, 9.7) | 0.258 | 3.4 (-2.4, 9.6) | 0.254 | 3.0 (-4.4, 11.0) | 0.436 | 1.8 (-4.2, 8.2) | 0.559 |
|  | v3 | 4.7 (-1.3, 11.0) | 0.128 | 4.7 (-1.3, 11.1) | 0.125 | 4.8 (-1.3, 11.3) | 0.124 | 4.8 (-1.2, 11.3) | 0.121 | 4.7 (-1.3, 11.1) | 0.127 | 4.4 (-2.6, 11.8) | 0.226 | 3.1 (-3.0, 9.6) | 0.321 |
|  | v4 | -12.3 (-17.3, -6.9) | < 0.001 | -12.3 (-17.4, -6.9) | < 0.001 | -12.3 (-17.4, -6.9) | < 0.001 | -12.1 (-17.3, -6.6) | < 0.001 | -12.3 (-17.4, -6.9) | <0.001 | -12.2 (-17.4, -6.8) | < 0.001 | -11.5 (-16.6, -6.1) | < 0.001 |

Abbreviations: DBP, diastolic blood pressure; MAP, mean arterial pressure; ca-PP, central aortic BP; ba-PP, brachial artery pulse pressure; SV, stroke volume; HR, heart rate; SEVR, subendocardial viability ratio; PWV, pulse wave velocity.

^a^ Shown as percent differences in the levels of health indices compared to the first (baseline) clinical visit.

Model 1 (main model) was adjusted for age, sex, and body mass index.

Model 2 = Model 1 + smoking status (current smokers vs. never smokers).

Model 3 = Model 1 + oxygen therapy (yes vs. no).

Model 4 = Model 1 + altitude sickness medication (yes vs. no).

Model 5 = Model 1 + the time from participants’ last clinical visit to their return to sea level.

Model 6 = Model 1 + HR. The dependent variable of this model is PWV.

Model 7 = Model 1 + MAP. The dependent variable of this model is PWV.

**Table S5. Selected pathways that potentially mediate the haemodynamic effects posed by high altitude exposure.**

| Pathway | | Pathway ID ^a^ | No. of genes ^b^ | Description ^c^ | Downstream haemodynamic modulations | Key genes and their functions ^d^ |
| --- | --- | --- | --- | --- | --- | --- |
| Vasoactive substance synthesis | Catecholamines | KEGG: hsa_M00042 | 5/2 | (1) Catecholamines (dopamine, norepinephrine, and epinephrine) are important neurotransmitters in peripheral and central nerve systems. Their functions on haemodynamics involve blood pressure, vasoconstriction and vasodilation of blood vessels, heart rate and cardiac tone, as well as arterial stiffness [16, 17].  (2) Short-term high-altitude exposure could induce increased levels of norepinephrine and epinephrine [18, 19]. | (1) Blood pressure;  (2) Cardiac function;  (3) Arterial stiffness. | (1) ***DBH***: This gene encodes dopamine beta-hydroxylase, which catalyzes the conversion of dopamine to norepinephrine; (2) ***PNMT***: This gene encodes phenylethanolamine N-methyltransferase, which catalyzes the conversion of norepinephrine to epinephrine; (3) *DDC*: This gene encodes dopa decarboxylase, which catalyzes the conversion of L-DOPA to dopamine. |
|  | Renin-angiotensin system (RAS) | KEGG: hsa04614 | 23/14 | (1) In the classical RAS, activation of type 1 angiotensin II receptor could increase sympathetic nervous system tone, blood pressure, vasoconstriction, cardiac hypertrophy, reactive oxygen species production and arterial stiffness, while decreasing nitric oxide synthesis, natriuresis, parasympathetic nervous system tone, and baroreflex sensitivity [20, 21].  (2) Telmisartan (an angiotensin II receptor blocker) treatment could attenuate high altitude-induced increases in systolic and diastolic blood pressure levels [19]. | (1) Blood pressure;  (2) Cardiac function;  (3) Arterial stiffness. | (1) ***CTSB***: This gene encodes cathepsin B, which catalyzes the conversion of prorenin to renin; (2) *REN*: This gene encodes renin, which catalyzes the conversion of angiotensinogen to angiotensin I; (3) ***ACE***, ***CTSG***, *CMA1*: These gene encode angiotensin I converting enzyme, cathepsin G, and chymase 1, respectively, which catalyze the conversion of angiotensin I to angiotensin II. |
|  | Endothelin-1 | WikiPathways: WP2197 | 33/21 | (1) Endothelin-1 is a potent vasoconstrictor produced by vascular endothelial cells. It has been demonstrated to increase blood pressure, cardiac contractility, arterial stiffness, sodium reabsorption and reactive oxygen species production, and decrease nitric oxide synthesis and vasodilation [22].  (2) Short-term high-altitude exposure could induce increased level of endothelin-1; bosentan (an mixed endothelin receptor A and B antagonist) treatment could lower high altitude-induced increase in the level of systolic pulmonary artery pressure [23]. | (1) Blood pressure;  (2) Cardiac function;  (3) Arterial stiffness. | ***EDN1***: This gene encodes endothelin-1. |
|  | Nitric oxide | WikiPathways: WP1995 | 8/1 | (1) Nitric oxide is critical in cardiovascular homeostasis. It is involved in several physiological processes such as vascular relaxation, anti-oxidative stress, excitation–contraction coupling in cardiac myocytes, and anti-arterial stiffening [24, 25].  (2) Short-term high-altitude exposure could initially decrease nitric oxide content in the body and then significantly increase its level [26]. | (1) Blood pressure;  (2) Cardiac function;  (3) Arterial stiffness. | ***NOS3***: This gene encodes nitric oxide synthase 3, which catalyzes the conversion of L-arginine to nitric oxide in endothelium. |
|  | Natriuretic peptides;  Calcitonin gene-related peptides | KEGG: hsa04270 | 134/90 | (1) There are 3 major natriuretic peptides, that are, atrial natriuretic peptide (ANP), brain natriuretic peptide (BNP), and C-type natriuretic peptide (CNP). The ANP and BNP are released primarily from the heart but circulate as hormones to act in various tissues in the body and induce vasodilation, natriuresis, and diuresis. CNP is derived form endothelial cells and myocardial tissue and might protect against postinfarction remodeling [27].  Calcitonin gene-related peptides are potent vasodilators released from sensory nerves and are suggested to have cardiovascular protective effects [28].  (2) Short-term high-altitude exposure could induce increases in atrial natriuretic peptide and brain natriuretic peptide [29].  It was demonstrated that short-term high-altitude exposure did not induce significant changes in calcitonin gene-related peptides [30, 31]. | Blood pressure. | (1) ***NPPA***, *NPPB*, *NPPC*: The genes encode atrial natriuretic peptide, brain natriuretic peptide, and C-type natriuretic peptide, respectively; (2) ***ADM***, ***ADM2***, *CALCA*, *CALCB*: The genes encode calcitonin gene-related peptides (potent vasodilators), with the first two genes encoding adrenomedullin and the last two genes encoding calcitonin gene-related peptide alpha and beta, respectively; (3) *NPR1*, ***NPR2***: The genes encode the receptors for atrial natriuretic peptide and brain natriuretic peptide, and C-type natriuretic peptide, respectively; (4) ***CALCRL***, ***RAMP1***, *RAMP2*, ***RAMP3***: The genes encode receptors for adrenomedullin and calcitonin gene-related peptides. |
|  | Eicosanoids | KEGG: hsa00590 | 61/36 | (1) Eicosanoids were involved in endothelial and vascular smooth muscle cell function. For example, thromboxane A2 could induce vasoconstriction and platelet aggregation, while prostaglandin I2 and prostaglandin D2 could induce vasodilation [32].  (2) Aspirin (an inhibitor of prostaglandins and thromboxanes) could significantly prevent headache at high altitudes [33]. Short-term high-altitude exposure could induce significant changes in the levels of eicosanoids [34]. | Blood pressure. | (1) ***TBXAS1***: This gene encodes thromboxane A synthase 1, which catalyzes the conversion of prostaglandin H2 to thromboxane A2; (2) *PTGIS*: This gene encodes prostaglandin I2 synthase, which catalyzes the conversion of prostaglandin H2 to prostacyclin (prostaglandin I2); (3) ***PTGDS***, *HPGDS*: The genes encode prostaglandin D2 synthase, which catalyzes the conversion of prostaglandin H2 to prostaglandin D2; (4) ***PTGIR***: The gene encodes the receptor for prostacyclin (prostaglandin I2). |
| Vascular smooth muscle cell (VSMC) contraction | | KEGG: hsa04270 | 134/90 | (1) On contraction, VSMCs shorten, thereby decreasing the diameter of a blood vessel and increasing vascular resistance to regulate the blood flow and pressure. VSMC contraction is triggered by an increase in intracellular free calcium concentration, promoting calcium–calmodulin-mediated phosphorylation of the regulatory myosin light chains and actin–myosin cross-bridge formation with consequent vasoconstriction [35]. VSMC contraction could also have an impact on acute arterial stiffening [36].  (2) Short-term high-altitude exposure could significantly increase blood pressure and arterial stiffness [37]. | (1) Blood pressure;  (2) Arterial stiffness. | (1) ***CALM1***, ***CALM2***, ***CALM3***, *CALML3*, ***CALML4***, *CALML5*, ***CALML6***: The genes encode calmodulin (calcium binding proteins); (2) ***MYLK***, *MYLK2*, *MYLK3*, ***MYLK4***: The genes encode myosin light chain kinase, which is a calcium/calmodulin dependent enzyme; (3) ***MYL6***, ***MYL6B***, ***MYL9***: The genes encode myosin light-chain; (4) ***ACTA2***, *ACTG2*: The genes encode actin; (5) ***CALD1***: This gene encodes caldesmon 1, a calmodulin- and actin-binding protein that plays an essential role in the regulation of smooth muscle and nonmuscle contraction; (6) ***ITPR1***, ***ITPR2***, ***ITPR3***: The genes encode an intracellular receptor for inositol 1,4,5-trisphosphate. Upon stimulation by inositol 1,4,5-trisphosphate, this receptor mediates calcium release from the endoplasmic reticulum and subsequent VSMC contraction; (7) *ADRA1A*, *ADRA1B*, *ADRA1D*: The genes encode adrenergic alpha1 receptors, which are involved in neurotransmission and regulate the sympathetic nervous system through binding of norepinephrine and epinephrine; (8) *AGTR1*: The gene encodes the receptor for angiotensin II (angiotensin II receptor type 1); (9) *EDNRA*: The gene encodes the receptor for endothelin-1 (endothelin receptor type A). |
| Cardiac muscle contraction | | KEGG: hsa04260 | 87/55 | (1) The cardiac muscle is responsible for the contractility of the heart and, therefore, the pumping action. The cardiac muscle must contract with enough force and enough blood to supply the metabolic demands of the entire body. This concept is termed cardiac output and is defined as the product of heart rate and stroke volume [38]. Cardiac muscle contraction is triggered by an increase in intracellular free calcium concentration. Binding of calcium to troponin C leads to a series of conformational rearrangements in troponin I and troponin T, which reveals strong myosin binding sites on actin, allowing muscle contraction [39].  (2) Short-term high-altitude exposure could induce higher levels of stroke volume and cardiac output; after several days’ sojourn at high altitude, the levels of stroke volume and cardiac output were reduced [40]. | Cardiac function. | (1) ***MYL4***, *MYL2*, *MYL3*: The genes encode myosin light chain; (2) *ACTC1*: The gene encodes actin; (3) ***TPM1***, ***TPM2***, ***TPM3***, ***TPM4***: The genes encode a tropomyosin, which functions in association with the troponin complex to regulate the calcium-dependent interaction of actin and myosin during muscle contraction; (4) ***TNNC1***, *TNNT2*, *TNNI3*: The genes encode Troponin C, Troponin T, and Troponin I, respectively. The binding of calcium to troponin C abolishes the inhibitory action of Troponin I, thus allowing the interaction of actin with myosin, the hydrolysis of ATP, and the generation of tension; (5) ***ATP2A1***, ***ATP2A2***, ***ATP2A3***: The genes encode SERCA Ca(2+)-ATPases, which are intracellular Ca(2+) pumps located in the sarcoplasmic reticula of muscle cells; (6) ***RYR2***, ***ASPH***, *CASQ2*, *TRDN*: The genes encode a Ca(2+) channel that mediates the release of Ca(2+) from the sarcoplasmic reticulum into the cytoplasm and thereby plays a key role in triggering cardiac muscle contraction; (7) ***ADRB1***, ***ADRB2***: The genes encode beta-adrenergic receptors. |
| Oxidative stress response | | KEGG: hsa05208 | 223/176 | (1) Reactive oxygen species could decrease nitric oxide production and antioxidant reserve, and induce oxidative modification of cardiovascular tissues and mitochondrial oxidative stress, resulting in vascular and cardiac dysfunction [41].  (2) Short-term high-altitude exposure could cause increased oxidative molecules (e.g., malondialdehyde and 8-hydroxyguanosine) and decreased antioxidatnt molecules (e.g., superoxide dismutase and glutathione peroxidase) [42]. | (1) Blood pressure;  (2) Cardiac function;  (3) Arterial stiffness. | (1) ***CYBA***, *NOX1*, *NOX4*, ***NCF1***, ***NCF2***: The genes encode the subunits of NADPH oxidase; (2) ***SOD1***, ***SOD2***, *SOD3*: The genes encode superoxide dismutase; (3) ***GPX1***, ***GPX3***, ***GPX4***, ***GPX7***: The genes encode glutathione peroxidase; (4) ***TXN***, ***TXN2***: The genes encode thioredoxin; (5) ***CAT***: This gene encodes catalase. |
| Aldosterone-mediated sodium reabsorption | | KEGG: hsa04960 | 37/24 | (1) Aldosterone could increase sodium reabsorption (coupled with water reabsorption and higher blood volume and pressure) in the distal nephron via activation of the apical epithelial sodium channel and the basolateral Na^+^,K^+^-ATPase [43].  (2) Short-term high-altitude exposure caused in initial decrease of aldosterone level, followed by an increase [44]. | Blood pressure. | (1) ***NR3C2***: This gene encodes the mineralocorticoid receptor, which mediates aldosterone actions on salt and water balance within restricted target cells; (2) ***NEDD4L***: The gene encodes E3 ubiquitin-protein ligase, which mediates the ubiquitination of multiple target substrates and plays a critical role in epithelial sodium transport by regulating the cell surface expression of the epithelial sodium channel; (3) ***SCNN1A***, *SCNN1B*, *SCNN1G*: The genes encode alpha, beta, and gamma subunit of the epithelial sodium channel, which mediates the electrodiffusion of the luminal sodium (and water, which follows osmotically) through the apical membrane of epithelial cells, and plays an essential role in electrolyte and blood pressure homeostasis; (4) *CYP11B2*: The gene encodes aldosterone synthase, which catalyzes the biosynthesis of adrenal mineralocorticoid aldosterone. |
| Carbonic anhydrase pathway | | Reactome: R-HSA-1480926 | 8/6 | (1) Carbonic anhydrase is responsible for reabsorption of dicarbonate, which is coupled with sodium and water reabsorption and thus causing increased blood volume [44]. Inhibiting carbonic anhydrase activity have potential vasodilating effect [45].  (2) Acetazolamide (a carbonic anhydrase inhibitor) treatment could lower the acute effects on blood pressure, arterial stiffness, and imbalance between myocardial oxygen supply and demand posed by high altitude [46, 47]. | (1) Blood pressure;  (2) Cardiac function;  (3) Arterial stiffness. | (1) ***CA1***, ***CA2***, ***CA4***: The genes encode carbonic anhydrase 1 and carbonic anhydrase 2, which catalyze the reversible hydration of carbon dioxide; (2) ***SLC4A1***: This gene encodes anion exchange protein 1, which mediates chloride-bicarbonate exchange in the kidney, and is required for normal acidification of the urine; (3) ***AQP1***: The gene encodes aquaporin 1, which is a water-specific channel that provides the plasma membranes of red cells and kidney proximal tubules with high permeability to water, thereby permitting water to move in the direction of an osmotic gradient; (4) ***RHAG***: This gene encodes Rh associated glycoprotein, which is a membrane channel that transports ammonium and carbon dioxide across the blood cell membrane. |

^a^ We referred to the pathways and the involved genes in Kyoto Encyclopedia of Genes and Genomes (KEGG) (https://www.kegg.jp/kegg/), WikiPathways (<https://www.wikipathways.org/>), and Reactome (https://reactome.org/).

^b^ The first number indicates the number of genes included in the pathway, and the second number indicates the number of genes (with a detection rate greater than 50%) included in statistical analysis. Some genes are shared by different pathways.

^c^ Two parts of information were involved in the description, that are, the general physiologic functions as well as the involvement in high altitude-induced haemodynamic changes, of the selected pathways.

^d^ Genes with bold font were included in statistical analysis because they have a detection rate of greater than 50%. We referred to the function of genes in GeneCards (<https://www.genecards.org/>), the database that provides comprehensive information on all annotated and predicted human genes.

**Table S6. Adaptive responses (activation vs. counterbalance) of the haemodynamics-regulating pathways to high altitude exposure (red and blue circles indicate upregulation and downregulation of genes, while circle number indicates magnitude of the changes).**

| Pathway | Activation ^a^ | | | | Counterbalance ^a^ | | | | Downstream haemodynamics ^b^ | | |
| --- | --- | --- | --- | --- | --- | --- | --- | --- | --- | --- | --- |
|  | Gene | v2 | v3 | v4 | Gene | v2 | v3 | v4 | Blood pressure | Cardiac function | Arterial stiffness |
| Vasoactive substance synthesis | *PNMT* |  |  |  | *NPPA* |  |  |  | √ | √ | √ |
|  | *CTSG* |  |  |  | *PTGDS* |  |  |  |  |  |  |
|  | *CTSB* |  |  |  | *PTGIR* |  |  |  |  |  |  |
|  | *EDN1* |  |  |  | *RAMP1* |  |  |  |  |  |  |
|  |  |  |  |  | *RAMP3* |  |  |  |  |  |  |
| VSMC contraction | *MYL6* |  |  |  | *CALM1* |  |  |  | √ |  | √ |
|  | *MYL6B* |  |  |  | *ITPR1* |  |  |  |  |  |  |
|  | *MYL9* |  |  |  | *ITPR2* |  |  |  |  |  |  |
|  | *ACTA2* |  |  |  | *ITPR3* |  |  |  |  |  |  |
|  | *CALD1* |  |  |  |  |  |  |  |  |  |  |
|  | *CALM2* |  |  |  |  |  |  |  |  |  |  |
|  | *CALM3* |  |  |  |  |  |  |  |  |  |  |
| Cardiac muscle contraction | *MYL4* |  |  |  | *TPM3* |  |  |  |  | √ |  |
|  | *TNNC1* |  |  |  | *ATP2A2* |  |  |  |  |  |  |
|  | *TPM1* |  |  |  | *ASPH* |  |  |  |  |  |  |
|  | *TPM2* |  |  |  |  |  |  |  |  |  |  |
| Oxidative stress signaling | *CYBA* |  |  |  | *GPX1* |  |  |  | √ | √ | √ |
|  | *NCF1* |  |  |  | *GPX4* |  |  |  |  |  |  |
|  | *GPX7* |  |  |  | *TXN* |  |  |  |  |  |  |
|  | *GPX3* |  |  |  | *TXN2* |  |  |  |  |  |  |
|  | *SOD2* |  |  |  | *SOD1* |  |  |  |  |  |  |
| ALD-mediated sodium reabsorption |  |  |  |  | *NEDD4L* |  |  |  | √ |  |  |
|  |  |  |  |  | *NR3C2* |  |  |  |  |  |  |
| Carbonic anhydrase pathway | *CA1* |  |  |  |  |  |  |  | √ | √ | √ |
|  | *CA2* |  |  |  |  |  |  |  |  |  |  |
|  | *RHAG* |  |  |  |  |  |  |  |  |  |  |
|  | *SLC4A1* |  |  |  |  |  |  |  |  |  |  |
|  | *AQP1* |  |  |  |  |  |  |  |  |  |  |

^a^ Activation and counterbalance refer to gene expression changes anticipating to activate or counterbalance haemodynamic effects. Shown as changes in the gene expression levels compared to the baseline (v1). The *p* values were adjusted for multiple comparisons using the Benjamini–Hochberg method. Significant increases and decreases are denoted by solid red and blue circles, respectively. Nonsignificant changes are denoted by open circles. The number of circles indicates magnitude of the changes of gene expression levels at high altitude, i.e., changes denoted by two circles are greater than those denoted by one circle (*p* < 0.05). Please refer to Table S5 for the function of genes.

^b^ Linking of the pathways to the downstream haemodynamic modulations is based on previous studies, as shown in detail in Table S5.

Abbreviations: VSMC, vascular smooth muscle cell; ALD, aldosterone.


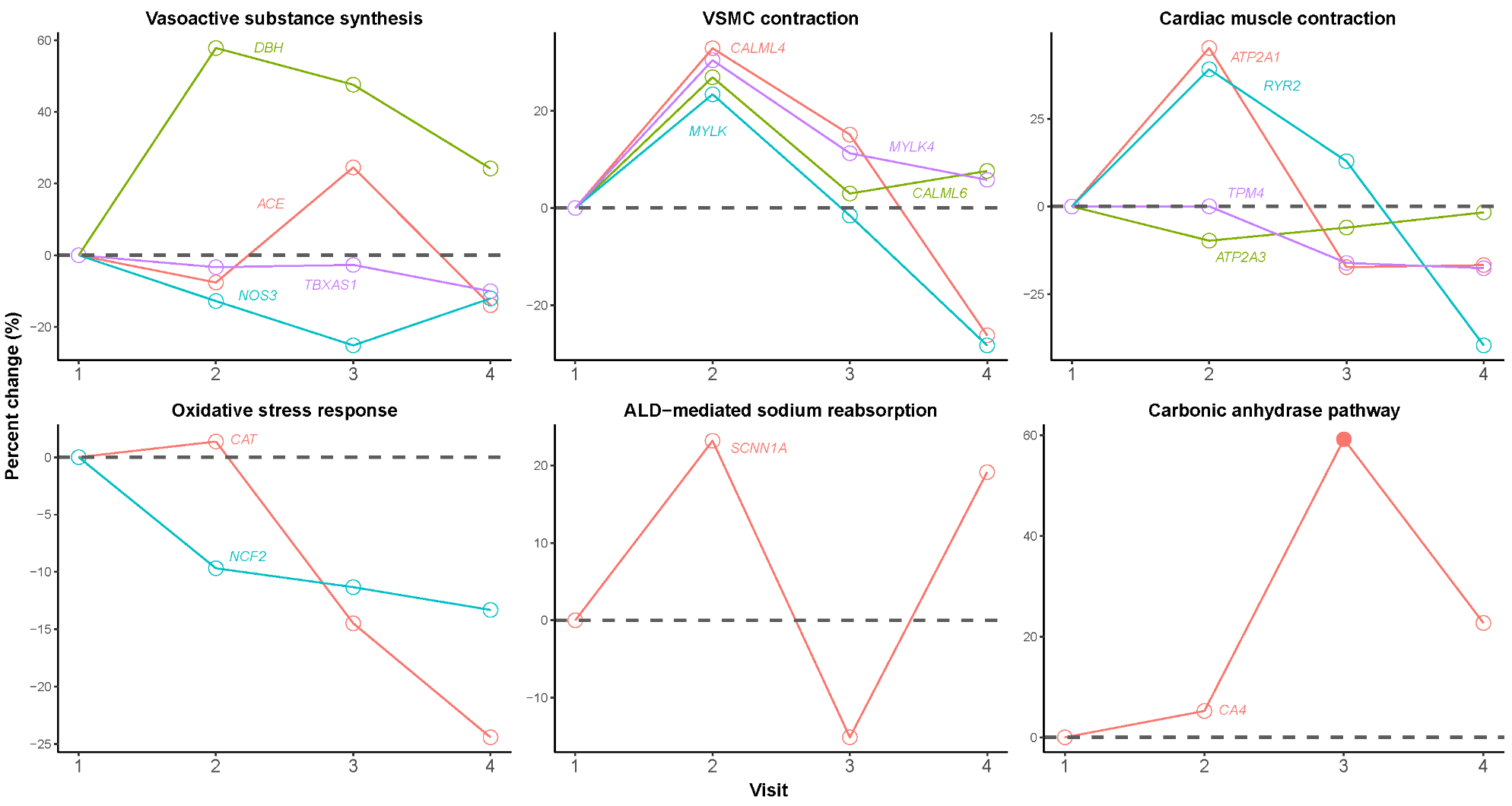


**Fig. S1. Changes in the key genes at four clinic visits.** Results were obtained from linear mixed-effects models with random intercepts of participants and adjusted for age, sex, and body mass index. All estimates are reported as percentage differences compared with the first (baseline) clinic visit. Significant (FDR_B-H_ <0.05) and non-significant differences are shown in solid and open circles, respectively. The pathways include vasoactive substance synthesis, VSMC contraction, cardiac muscle contraction, oxidative stress response, ALD-mediated sodium reabsorption, and carbonic anhydrase pathway. Gene names are expressed in italics.

Abbreviation: VSMC, vascular smooth muscle cell contraction; ALD, aldosterone.


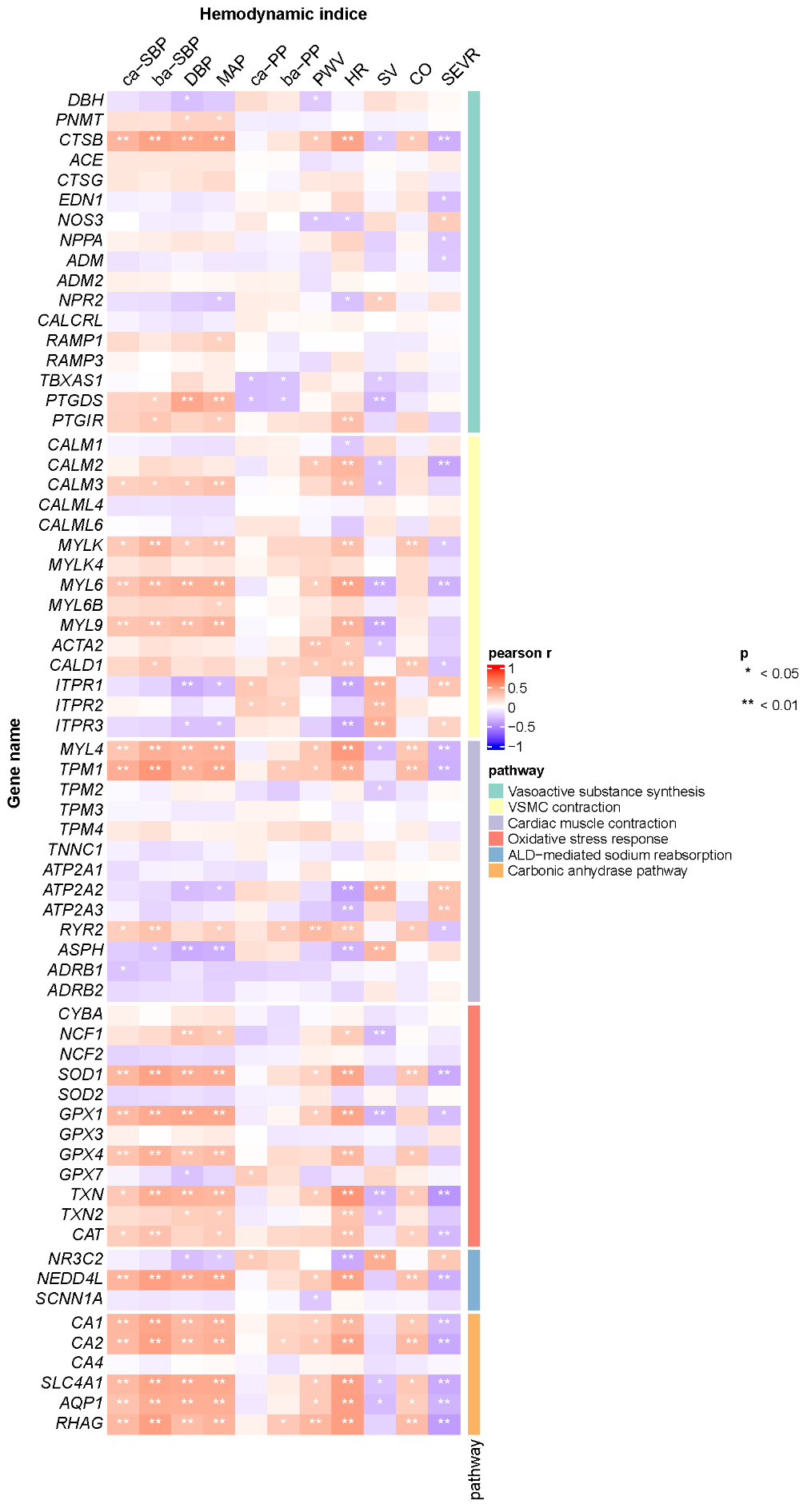


**Fig. S2. Correlations between haemodynamic indices and key genes involved in haemodynamics-regulating pathways.** The results were obtained by Pearson correlation. Significant correlation with a *p*-value <0.05 was denoted as asterisk. The pathways include vasoactive substance synthesis, oxidative stress response, VSMC contraction, cardiac muscle contraction, carbonic anhydrase pathway, and aldosterone-mediated sodium reabsorption. Gene names are expressed in italics.

Abbreviations: PWV, pulse wave velocity; ca-SBP, central aortic systolic blood pressure; ba-SBP, brachial artery systolic blood pressure; DBP, diastolic blood pressure; ca-PP, central aortic pulse pressure; ba-PP, brachial artery pulse pressure; MAP, mean arterial pressure; SV, stroke volume; HR, heart rate; CO, cardiac output; SEVR, subendocardial viability ratio; VSMC, vascular smooth muscle cell contraction; ALD, aldosterone.

**Statistical Analysis Code**

setwd("C:\\Users\\Xu\\Desktop\\2022\\HA ")

data_1=read.csv("data.csv")

library("lme4")

library("lmerTest")

library("splines")

Index_start=90

Index_end=157

Index_nvar=Index_end-Index_start+1

p_1=rep(NA,Index_nvar)

effect_1= rep(NA, Index_nvar)

effect_low_1= rep(NA, Index_nvar)

effect_high_1= rep(NA, Index_nvar)

z_1=rep(NA, Index_nvar)

se_1= rep(NA, Index_nvar)

p_2=rep(NA,Index_nvar)

effect_2= rep(NA, Index_nvar)

effect_low_2= rep(NA, Index_nvar)

effect_high_2= rep(NA, Index_nvar)

z_2=rep(NA, Index_nvar)

se_2= rep(NA, Index_nvar)

p_3=rep(NA,Index_nvar)

effect_3= rep(NA, Index_nvar)

effect_low_3= rep(NA, Index_nvar)

effect_high_3= rep(NA, Index_nvar)

z_3=rep(NA, Index_nvar)

se_3= rep(NA, Index_nvar)

Index_variable= rep(NA, Index_nvar)

number=1

for(j in Index_start:Index_end){

Index=colnames(data_1)[j]

m=lmer(get(Index)~factor(period)+(1|ID)+age+sex+BMI,data=data_1,na.action=na.omit)

beta1=summary(m)$coefficients[2,1]

se1= summary(m)$coefficients[2,2]

z1=(beta1/se1)

p1=2*(1-pnorm(abs(z1)))

effect1=(exp(beta1))-1

low_1=exp((beta1-1.96*se1))-1

high_1=exp((beta1+1.96*se1))-1

beta2=summary(m)$coefficients[3,1]

se2= summary(m)$coefficients[3,2]

z2=(beta2/se2)

p2=2*(1-pnorm(abs(z2)))

effect2=(exp(beta2))-1

low_2=exp((beta2-1.96*se2))-1

high_2=exp((beta2+1.96*se2))-1

beta3=summary(m)$coefficients[4,1]

se3= summary(m)$coefficients[4,2]

z3=(beta3/se3)

p3=2*(1-pnorm(abs(z3)))

effect3=(exp(beta3))-1

low_3=exp((beta3-1.96*se3))-1

high_3=exp((beta3+1.96*se3))-1

p_1[number]=as.numeric(p1)

effect_1[number]=as.numeric(effect1)

effect_low_1[number]=as.numeric(low_1)

effect_high_1[number]=as.numeric(high_1)

z_1[number]=as.numeric(z1)

se_1[number]=as.numeric(se1)

p_2[number]=as.numeric(p2)

effect_2[number]=as.numeric(effect2)

effect_low_2[number]=as.numeric(low_2)

effect_high_2[number]=as.numeric(high_2)

z_2[number]=as.numeric(z2)

se_2[number]=as.numeric(se2)

p_3[number]=as.numeric(p3)

effect_3[number]=as.numeric(effect3)

effect_low_3[number]=as.numeric(low_3)

effect_high_3[number]=as.numeric(high_3)

z_3[number]=as.numeric(z3)

se_3[number]=as.numeric(se3)

Index_variable[number]=Index

number=number+1

}

result_data_1=as.data.frame(cbind(Index_variable,p_1,z_1,se_1,effect_1,effect_low_1,effect_high_1,p_2,z_2,se_2,effect_2,effect_low_2,effect_high_2,p_3,z_3,se_3,effect_3,effect_low_3,effect_high_3))

write.csv(result_data_1,file="result.csv")
